# TTSwing: a Dataset for Table Tennis Swing and Racket Kinematics Analysis

**DOI:** 10.1038/s41597-025-04680-y

**Published:** 2025-02-26

**Authors:** Che-Yu Chou, Zheng-Hao Chen, Yung-Hoh Sheu, Hung-Hsuan Chen, Min-Te Sun, Sheng K. Wu

**Affiliations:** 1 Department of Computer Science and Information Engineering, National Central University, Taoyuan, 320317 Taiwan; 2 Department of Computer Science and Information Engineering, National Formosa University, Yunlin, 63200 Taiwan; 3 Department of Sport Performance, National Taiwan University of Sport, Taichung, 404401 Taiwan

**Keywords:** Health care, Engineering

## Abstract

We introduce TTSwing, a novel dataset designed to analyze table tennis swings. The dataset was collected using custom racket grips embedded with 9-axis motion sensors, which provide precise kinematic data on swings. In addition, we provide anonymized demographic data for players. The dataset was collected from 93 participants, all of whom are elite table tennis players from Taiwan. We detail the data collection and annotation procedures. These data are expected to improve the understanding of player performance and facilitate the development of tailored training programs and biomechanical analyses, offering practical benefits to both athletes and coaches. TTSwing has excellent potential to facilitate innovative research in table tennis analysis and is a valuable resource for the scientific community. We release the dataset and the experimental codes for reproducibility.

## Background & Summary

Since its inclusion as an Olympic sport in 1988, table tennis has gained widespread popularity. It is enjoyed around the world as a competitive sport and a common recreational pastime among players of all levels and ages. Meanwhile, with advances in sensor technologies and machine learning algorithms, there is increasing interest in using data-driven approaches to analyze sports, including table tennis. Such approaches provide valuable insight into player performance and inform training programs for players.

This paper introduces the TTSwing (Table Tennis Swing) dataset, a novel dataset that includes swing information collected by the 9-axis sensors embedded in the grips of customized paddles. The swing here refers to the powerful forehand smash movement. Since the sensor is embedded in the handler of a racket, perhaps a more precise term for swing information is racket kinematics. We use these two terms interchangeably in the following. TTSwing dataset accurately details racket kinematics, offering critical insights into player performance, shot quality, and technical differentiation among skill levels^1–3^. In addition to swing information, the dataset includes anonymized demographic details of players, such as age, gender, height, weight, racket-holding hand, and years of experience in the game. Combined with kinematic data, these demographic attributes allow for an in-depth analysis of the interplay between player characteristics and performance metrics, enabling the development of customized training programs and biomechanical studies. This comprehensive dataset provides a valuable resource for studying table tennis swings and can be used to develop new techniques and approaches to improve table tennis skills.

Previous studies have taken advantage of advances in sensor technology to collect detailed information on athletes or user movements^4–6^. For example, researchers attached a DELSYS sensor to ten points in the right arm to collect muscle information^7^. They also analyzed the differences between professional and amateur players when stroking. A follow-up paper expanded the collected data by placing a hang3.0 sensor in nine different areas, e.g., hands, limbs, and waists^8^. The authors classified motions according to acceleration and angular velocity and used the results to improve the stroke posture of the players. Some studies have placed the smart device on the wrist of the player to collect acceleration and angular velocity data during stroke^9–11^. However, using excessive portable devices may make player movements less natural and may indirectly impact the player’s performance. Another work embedded sensors in the grip of the racket^12^ and predicted the spin of table tennis and stroke type^13,14^. Compared to other collection methods, embedding sensors into the grip reduces the burden on the player and makes data collection easier. In addition, the embedded sensor in the grip captures the racket movement, allowing the detection of even subtle changes in racket movement. In our paper, we have chosen to utilize the last method for data collection. However, previous studies only collected swing information from a limited number of players, typically ranging from a few to a dozen^12–14^. In contrast, our dataset is a unique resource for future research, as we collected and analyzed information from nearly 100 elite players, providing a much larger sample size than in previous experiments.

In summary, our work has the following contributions.

- We present a new dataset, TTSwing, that captures professional table tennis players’ swings (racket kinematics) along with anonymized demographic information of players. We describe the collection and annotation process. To our knowledge, this is the largest open dataset for professional players’ swing information with their anonymized demographic information^12– 14^.
- The dataset provides a high-resolution record of racket movement, enabling future research in table tennis analytics, biomechanics, and player performance assessment. By offering structured and well-annotated swing data, TTSwing can serve as a valuable resource for studies on skill evaluation, training optimization, and data-driven sports science applications.
- To support reproducibility, we openly release the dataset together with structured documentation detailing the data attributes, the collection process, and potential use cases. This dataset lays a foundation for future research in table tennis swing analysis and broader applications in sports technology.

## Methods

### Ethics Approval and Consent to Participate

This work is approved by the Institutional Review Board of Jen-Ai Hospital in Dali, Taichung, Taiwan, under approval number 202200001B0. All participants signed an agreement form stating that the data obtained from the experiment can be published in academic journals, with an ID that replaces their names.

### Challenges of Collecting Swing Data

Collecting data on table tennis swings presents a variety of challenges. Commonly used methods, such as video recording or attaching sensors to the human body, have limitations. Video recording requires a fixed camera. It can be challenging to replicate the same environmental setup from one place to another, making it difficult to collect accurate data in different environments. Attaching sensors to the body using smartwatches, smartphones, or other sensors may influence players’ movements, creating interfering factors in the analysis. Embedding sensors into the equipment, such as the paddle, is a better option. However, previous studies that used this approach collected only data from a few players, typically no more than 20^12–14^. In addition, a significant workforce is required to split the continuous signals into stroke-based data.

Given these limitations, we develop a new method that addresses the challenges of collecting swing data from table tennis. We embed sensors directly into the racket to collect data from more than 90 professional players, generating a dataset of over 90,000 strokes. Our approach allows for accurately collecting stroke information without significantly affecting players’ performance. Additionally, we have developed an automated method to split the collected continuous waveform data into each stroking data, making data processing more efficient. By collecting data from many players, our method provides a more comprehensive understanding of the mechanics and nuances of table tennis swings. Our approach overcomes the limitations of previous studies, which only collected data from a few players. The resulting dataset opens up new possibilities for research and development in the field, allowing for the creation of more advanced applications and complex models.

### Hardware

**Fig. 1:**
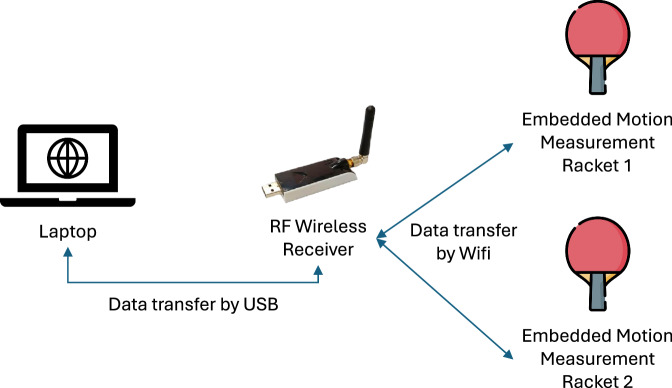
An overview of the entire system. The location of the sensor on the paddle is shown in Fig. 2.

Figure 1 gives an overview of the entire system. Motion sensors are embedded in racket grips to collect data. We use the shakehand grip style racket as it is more prevalent among players. The data collected are transferred to the RF wireless receiver, which transfers the information to a computer through a USB port.

**Fig. 2:**
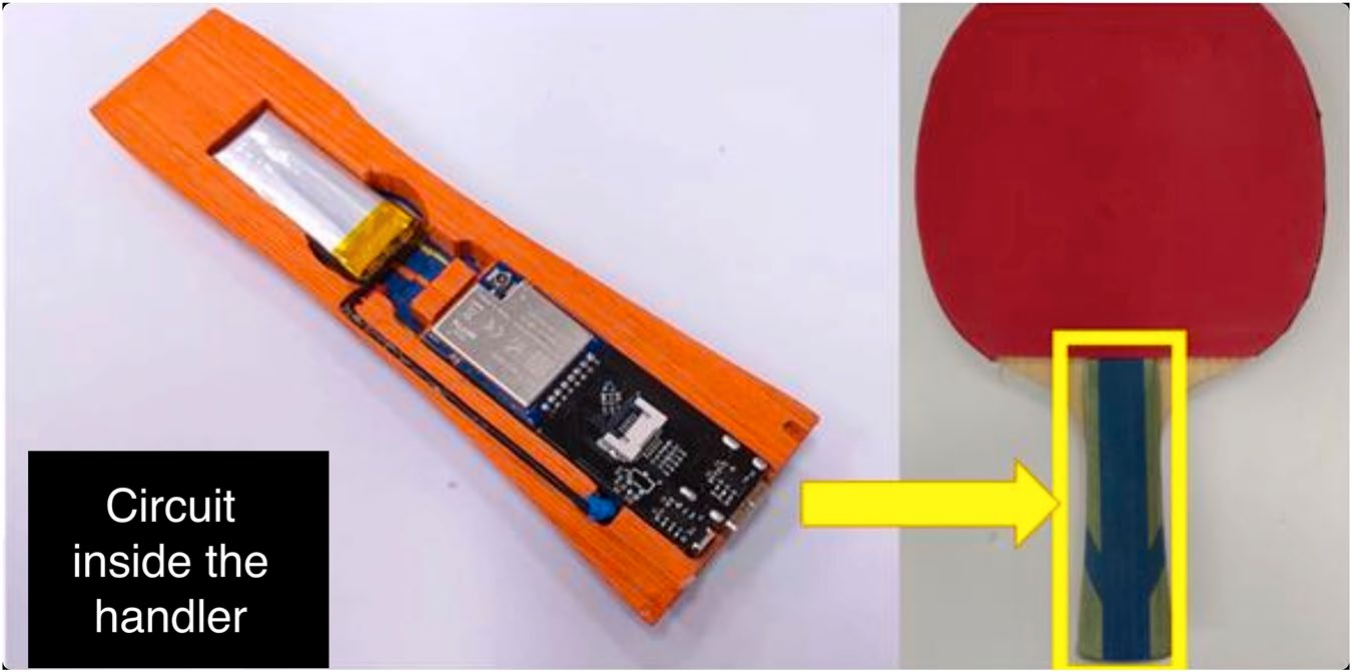
Embedding detection sensors and communication modules inside the paddle of a racket.

**Fig. 3:**
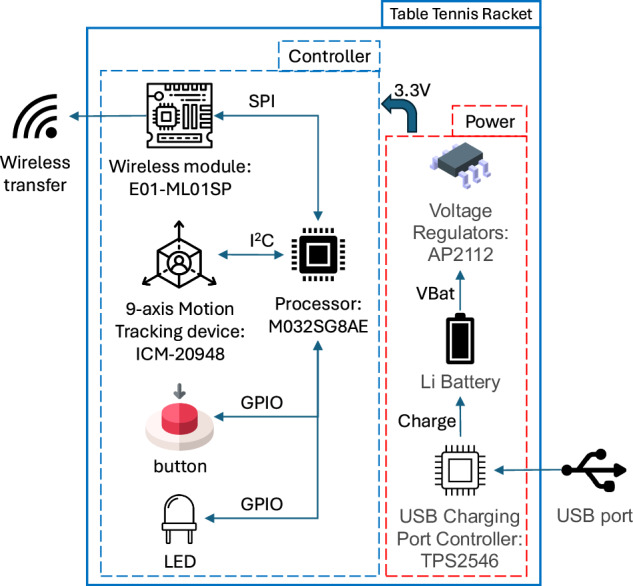
Hardware architecture of the sensor in the paddle of a racket.

Figure 2 shows how we embed the hardware in the table tennis racket. The embedded components include an inertial measurement unit (ICM-20948), a module for radio frequency (RF) wireless transmission (E01-ML01SP), and affiliated components such as the button and the RGB LED for simple I/O communications, as shown in Fig. 3. A lithium battery powers the system, and a TPS2546 USB charging port is connected to a 5V DC external power supply to maintain the battery’s power. Eventually, the embedded racket weighs approximately 190 grams, which falls within the weight range of a regular racket.

**Fig. 4:**
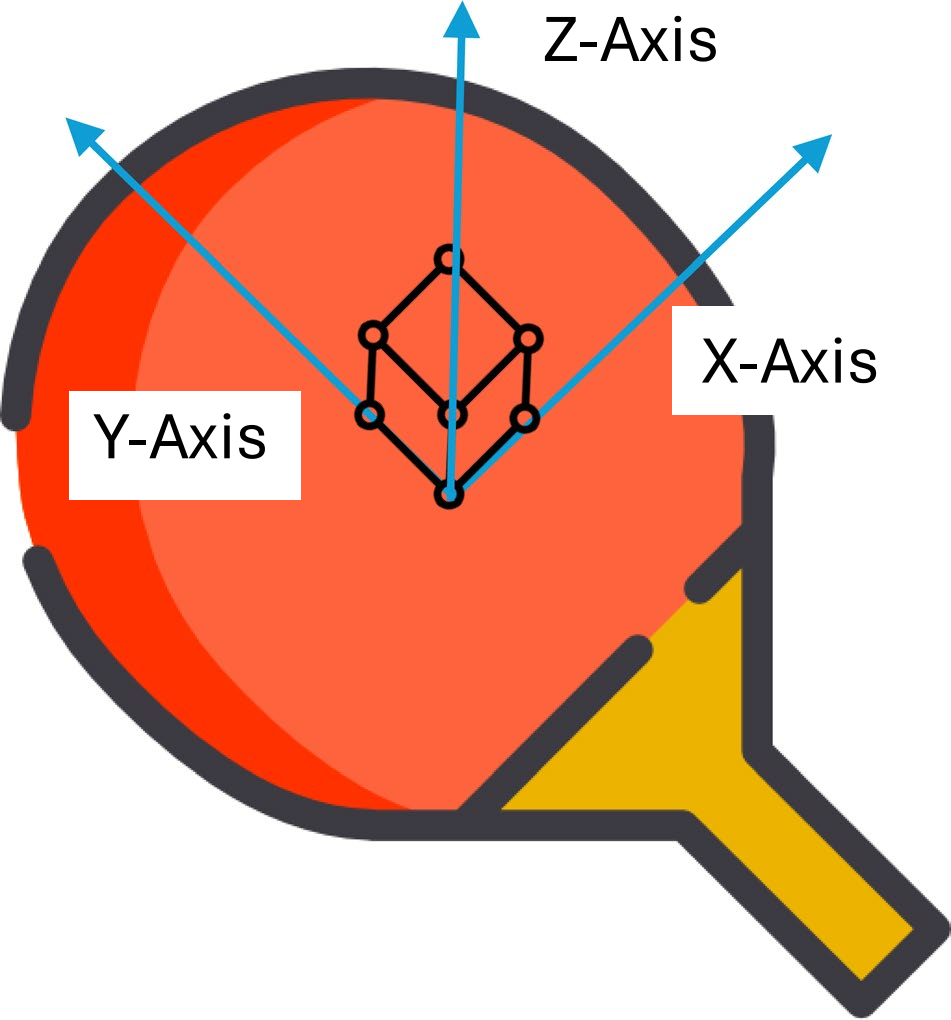
Definitions of the three axes.

The motion sensor, ICM-20948, is critical to collecting swing data. The sensor integrates a 3-axis accelerometer, a 3-axis gyroscope, and a 3-axis compass, forming a 9-axis sensor that can effectively measure 3-axis acceleration, 3-axis angular velocity, and 3-axis magnetic field data. The three axes are defined according to Fig. 4: the positive x-axis is to the right, the positive y-axis is forward, and the positive z-axis is perpendicular to the red side of the racket.

The motion sensors embedded in the racket grips are configured with a sampling rate of 80 Hz, ensuring sufficient temporal resolution to capture detailed kinematic data. Calibration is performed using a standard vibration exciter model 394C06 from PCB Piezotronics, ensuring the accuracy and reliability of the collected measurements.

### Swing Data Collection

We invited 93 Taiwanese players from Group A to participate in the data collection process. The players in Group A are elite players who have majored in physical education or have won medals in important competitions. Participants were asked to perform at least one of three different swing modes using the proposed racket. The three modes include swing in the air, full power stroke, and stable hitting. Each mode requires the participants to swing the racket 50 times continuously to generate a complete set of waveforms. In the full power stroke mode, the serving machine sets three ball speeds for players to hit.

### Split the Complete Waveform Set into Separated Stroke Waveforms

This section details the methodology for dividing a raw waveform into stroke-based waveforms. Before diving into the specifics, we summarize the basic approach: the segmentation method involves integrating multi-axis signals into a single waveform, normalizing it to remove inconsistencies and noise, and detecting peaks and troughs to isolate individual strokes. This approach, based on a combination of trend removal and peak detection, does not follow a prenamed standard but adopts principles from signal processing tailored to this dataset. Table 1 shows the pseudocode of the split process. We describe the details in the following.

**Table 1:** Waveform Split Algorithm.

Step	Description
1	Compute the integrated waveform using Equation (1).
2	Normalize the integrated waveform: a. Calculate the trend line by the combined signal. b. Subtract the trend line from the combined signal. c. Apply a low-pass filter to cutoff high frequencies. d. Scale the signals to the range [0, 1].
3	Segment the waveform: a. While the current number of intersection points × 2 is less than the total number of strokes, move down the horizontal line. b. Search for troughs to the right and left.
4	Split the original waveform by the troughs.
5	Return the waveform for each stroke.

**Fig. 5:**
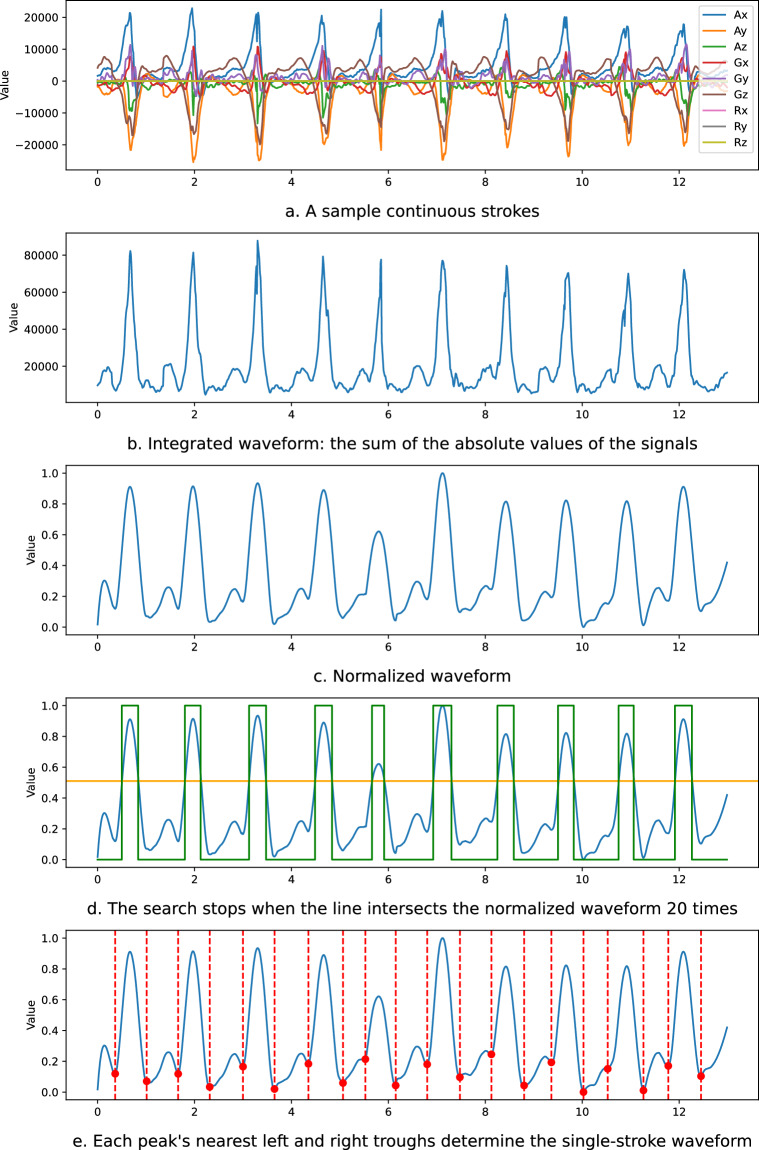
An example of finding the cutting points of the waveforms.

As mentioned above, each participant in the study swung the racket 50 times continuously, resulting in a complete set of waveforms. For further analysis, we want to divide each complete waveform set into 50 separate stroke waveforms. However, this proved challenging, as different strokes exhibit different strengths and trajectories, generating unique waveforms. Figure 5a illustrates a portion of a complete waveform set comprising ten consecutive strokes, each stroke waveform showing a similar but distinct shape.

First, we integrate the six waveforms from the accelerometer and gyroscope into a single *f*(*t*) by summing the absolute values of these waves, as shown by Equation (1).

1 $$f(t)=| {A}_{X}(t)| +| {A}_{Y}(t)| +| {A}_{Z}(t)| +| {G}_{X}(t)| +| {G}_{Y}(t)| +| {G}_{Z}(t)| ,$$

where *A*_*X*_(*t*), *A*_*Y*_(*t*), *A*_*Z*_(*t*) are the 3-axis values from accelerometer at time *t*, *G*_*X*_(*t*), *G*_*Y*_(*t*), *G*_*Z*_(*t*) are the values from gyroscope at *t*.

We call the outputted waveform the *integrated waveform*. Figure 5b displays an example of the integrated waveform.

We normalize the integrated waveform as follows. First, we remove the trend from the integrated waveform to remove the inconsistencies of each stroke from the same player. Next, we apply a low-pass filter provided by ICM-20948 to remove high-frequency noise. Finally, we scale the waveform to be within the range of 0 to 1. These steps help to speed up the peak detection process in the subsequent steps. Figure 5c shows an example of the normalized waveform.

We segment the normalized waveform by stroke based on the following steps. We first plot a horizontal line *y* = 1, which interacts with the peak of the entire normalized waveform. Since the number of swings is known, we gradually move the horizontal line downward until the number of intersection points equals twice the number of swings. For example, in Figure 5d, the number of known stroking features is 10, and the search is stopped when 20 intersection points are found. The peak of each stroke is expected to be within two neighboring intersections.

**Fig. 6:**
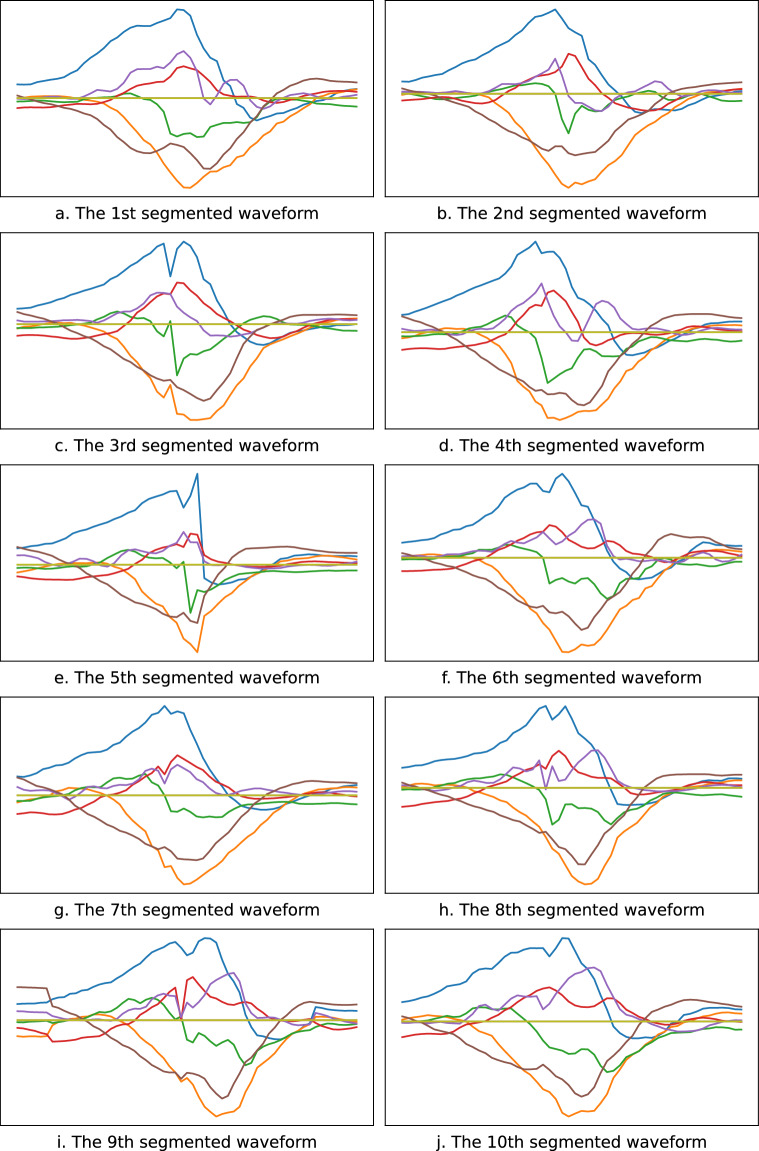
Samples of the segmented waveforms.

Based on the identified peaks, we further search for the nearest troughs to the left and right. These two troughs are split points for separating a complete stroke waveform. Figure 5e shows the two troughs found for each wave, and Fig. 6 shows the 10 segmentation results.

## Data Records

The dataset is available at Dryad^15^. This section introduces the released features and data statistics.

### Overview of the Released Data

The data released includes swing and personal features integrated into a tabular (CSV) format file. Table 2 shows the column headings.

**Table 2:** Field descriptions in the dataset.

Field Name	Description
id	Unique ID of the player
date	Swing test date
testmode	Represents three different tests, including ‘air swing’ (mode 0), ‘aerobic swing’ (mode 1), and ‘full-force swing’ (mode 2)
teststage	Represents three different ball speeds 1, 2, or 3. teststage is used only when testmode=1 (i.e., ‘aerobic swing’). When testmode is 0 or 2, the value of teststage is 0.
fileindex	Represents the *n*th test performed by the same participant under the same mode and stage conditions.
count	The *n*th swing/stroke of a test
ax_mean	Mean of X-axis acceleration (unit: LSB/G)
ay_mean	Mean of Y-axis acceleration (unit: LSB/G)
az_mean	Mean of Z-axis acceleration (unit: LSB/G)
gx_mean	Mean of X-axis angular velocity (unit: LSB/deg/s)
gy_mean	Mean of Y-axis angular velocity (unit: LSB/deg/s)
gz_mean	Mean of Z-axis angular velocity (unit: LSB/deg/s)
ax_var	Variance of X-axis acceleration (unit: the square of LSB/G)
ay_var	Variance of Y-axis acceleration (unit: the square of LSB/G)
az_var	Variance of Z-axis acceleration (unit: the square of LSB/G)
gx_var	Variance of X-axis angular velocity (unit: the square of LSB/deg/s)
gy_var	Variance of Y-axis angular velocity (unit: the square of LSB/deg/s)
gz_var	Variance of Z-axis angular velocity (unit: the square of LSB/deg/s)
ax_rms	Root mean square of X-axis acceleration (unit: LSB/G)
ay_rms	Root mean square of Y-axis acceleration (unit: LSB/G)
az_rms	Root mean square of Z-axis acceleration (unit: LSB/G)
gx_rms	Root mean square of X-axis angular velocity (unit: LSB/deg/s)
gy_rms	Root mean square of Y-axis angular velocity (unit: LSB/deg/s)
gz_rms	Root mean square of Z-axis angular velocity (unit: LSB/deg/s)
a_max	Maximum acceleration of a swing (unit: LSB/G)
a_mean	Mean acceleration of a swing (unit: LSB/G)
a_min	Minimum acceleration of a swing (unit: LSB/G)
g_max	Maximum angular velocity of a swing (unit: LSB/deg/s)
g_mean	Mean angular velocity of a swing (unit: LSB/deg/s)
g_min	Minimum angular velocity of a swing (unit: LSB/deg/s)
a_fft	Fast Fourier transform of acceleration (unit: LSB/G)
g_fft	Fast Fourier transform of angular velocity (unit: LSB/deg/s)
a_psd	Power spectral density of acceleration (unit: (LSB/G)^2^/Hz)
g_psd	Power spectral density of angular velocity (unit: (LSB/deg/s)^2^/Hz)
a_kurt	Kurtosis of acceleration (no unit)
g_kurt	Kurtosis of angular velocity (no unit)
a_skewn	Skewness of acceleration (no unit)
g_skewn	Skewness of angular velocity (no unit)
a_entropy	Spectral entropy of acceleration (no unit)
g_entropy	Spectral entropy of angular velocity (no unit)
gender	Player’s gender: 1 for males and 0 for females
age	Player’s age: high/medium/low
play years	Years of playing experience: high/medium/low
height	Player’s height: high/medium/low
weight	Player’s weight: high/medium/low
handness	Dominant hand (i.e., hand used in daily life): 1 for the right hand; 0 for the left hand
hold racket hand	Racket-holding hand: 1 for the right hand; 0 for the left hand

### Released Swing Features

Table 3 details the three types of features derived from the waveforms. The first type includes the mean, variance, and root mean square of the accelerations and angular velocities along the three axes (i.e., *A*_*X*_(*t*), *A*_*Y*_(*t*), *A*_*Z*_(*t*), *G*_*X*_(*t*), *G*_*Y*_(*t*), *G*_*Z*_(*t*)), which result in 18 features. The second type contains the mean, maximum value, minimum value, skewness, and kurtosis of the overall acceleration *A*(*t*) and angular velocity *G*(*t*), which generates ten features. Finally, we apply the Fourier transform to the acceleration and angular velocity waveforms and further derive the spectral density values and spectral entropy values for the acceleration and angular velocity, resulting in six features.

**Table 3:** A list of features extracted from a waveform.

Type	Input	Computation	Number of generated features
1	*A*_*x*_(*t*), *A*_*y*_(*t*), *A*_*z*_(*t*), *G*_*x*_(*t*), *G*_*y*_(*t*), *G*_*z*_(*t*)	mean, variance, root mean square	18
2	*A*(*t*), *G*(*t*)	mean, max, min, skewness, kurtosis	10
3	*A*(*t*), *G*(*t*)	Fourier Transform, spectral density, spectral entropy	6

By converting the continuous waveform signals into a finite number of features, it should be more convenient to apply various machine learning and deep learning models for further analyses.

The unit of the accelerations (e.g., *A*_*X*_(*t*), *A*_*Y*_(*t*), *A*_*Z*_(*t*)) is LSB/G (least significant bit per unit of G-force). By multiplying this value by 2/32768, the original G value can be obtained. The unit of angular velocities (e.g., *G*_*X*_(*t*), *G*_*Y*_(*t*), and *G*_*Z*_(*t*)) is LSB/deg/s (least significant bit per unit of angular velocity). By multiplying this value by 250/32768, the original DPS (degree per second) can be obtained.

### Released Personal Features

We provide anonymized personal information for each player, including gender, age, height, weight, handedness, racket-holding hand, and years of experience. These demographic details can be used for group comparisons, such as examining waveform characteristics across different groups of players based on factors such as gender, dominant hand, or skill level. Additionally, to prevent attackers from recognizing a player’s identity from unique numerical features, we categorized each numerical value into one of three labels – “low”, “medium”, or “high” – according to the feature’s distribution.

### Data Statistics

We recruited 93 players, comprising 53 males and 40 females, and 78 are right-handed while 15 are left-handed. The statistical summary for other numerical features is listed in Table 4.

**Table 4:** Statistical summary of players’ numerical features.

	age (year)	height (cm)	weight (kg)	BMI (kg/m^2^)	exp years (year)
Q1	13.78	159.0	48.00	18.67	6.00
Median	15.70	165.0	56.00	20.24	7.25
Mean	16.84	164.9	55.97	20.48	8.15
Q3	19.70	170.5	60.00	22.08	10.00

Minimum and maximum values are omitted to prevent deducing the players’ identities.

Based on the swings of the 93 players, we generate 97, 350 records: 7, 500 of them are mode 0 (swing in the air); 73, 850 of them are mode 1(full power stroke); 16, 000 of them are mode 2 (stable hitting).

## Technical Validation

The TTSwing dataset was collected under controlled conditions to ensure the precision and reliability of the recorded data. The embedded 9-axis motion sensors were pre-calibrated, ensuring precise accelerometer, gyroscope, and magnetometer readings. Furthermore, real-time wireless transmission of swing data to a laptop helped minimize possible data loss or corruption.

Demographic attributes such as gender, age, height, weight, and racket-holding hand were self-reported by participants, with verification when possible, such as verification from coaches or retired athletes. Since these attributes are inherently factual and do not involve subjective measurement, they can be considered ground truth within the dataset.

### Potential Sources of Error in Data Collection

Several potential sources of error could affect the data collection process. First, variations in sensor calibration could introduce inconsistencies in the measurements. Although embedded sensors were calibrated before data collection, slight drifts in sensitivity or accuracy may occur over time. Second, placement of the sensor in the racket grip, while designed to minimize interference, can result in slight deviations due to minor changes during long-term use or repetitive impacts. Third, while efforts were made to automate waveform segmentation, algorithmic errors during stroke identification may occasionally misclassify strokes or omit key features, particularly for players with unconventional playing styles.

## Usage Notes

We conduct experiments based on Python version 3.10. Packages and their tested versions are listed in Table 5.

**Table 5:** Python packages and tested versions to run the code.

package name	version number
numpy	1.22.3
pandas	1.4.2
sklearn	1.0.2
TensorFlow	2.8.3
keras	2.8.0
matplotlib	3.5.2
openpyxl	3.0.10
tqdm	4.65.0

Once the code is downloaded from the repository, the users can use pip install -r requirements.txt to reproduce the experimental environment. To run the code, users can change the directory to the src folder and run the Python scripts in the folder to reproduce the results.

## Data Availability

The code and data are available on Dryad at https://datadryad.org/stash/dataset/doi:10.5061/dryad.0zpc8677f^15^.
